# Single Intraperitoneal Injection of Monocrotaline as a Novel Large Animal Model of Chronic Pulmonary Hypertension in Tibet Minipigs

**DOI:** 10.1371/journal.pone.0078965

**Published:** 2013-11-11

**Authors:** Guang-qiao Zeng, Rong Liu, Hai-xing Liao, Xin-feng Zhang, Yuan-xin Qian, Bao-hua Liu, Qing-hong Wu, Jin Zhao, Wei-wang Gu, Hong-tao Li

**Affiliations:** 1 First Affiliated Hospital, Guangzhou Medical University; Guangzhou, Guangdong, China; 2 State Key Laboratory of Respiratory Diseases, Guangzhou Medical University, Guangzhou, Guangdong, China; 3 Laboratory Animal Centre, Southern Medical University, Guangzhou, Guangdong, China; VU University Medical Center, The Netherlands

## Abstract

**Objective:**

The purpose of this study was to establish an animal model of chronic pulmonary hypertension with a single*-*dose intraperitoneal injection of monocrotaline (MCT) in young Tibet minipigs, so as to enable both invasive and noninvasive measurements and hence facilitate future studies.

**Methods:**

Twenty-four minipigs (8*-*week*-*old) were randomized to receive single*-*dose injection of 12.0 mg/kg MCT (MCT group, n = 12) or placebo (control group, n = 12 each). On day 42, all animals were evaluated for pulmonary hypertension with conventional transthoracic echocardiography, right heart catheterization (RHC), and pathological changes. Findings of these studies were compared between the two groups.

**Results:**

At echocardiography, the MCT group showed significantly higher pulmonary arterial mean pressure (PAMP) compared with the controls (*P*<0.001). The pulmonary valve curve showed v-shaped signals with reduction of a-waves in minipigs treated with MCT. In addition, the MCT group had longer pulmonary artery pre*-*ejection phases, and shorter acceleration time and ejection time. RHC revealed higher mean pulmonary arterial pressure (mPAP) in the MCT group than in the control group (*P*<0.01). A significant and positive correlation between the mPAP values and the PAMP values (R = 0.974, P<0.0001), and a negative correlation between the mPAP and ejection time (R = 0.680, P<0.0001) was noted. Pathology demonstrated evidence of pulmonary vascular remodeling and higer index of right ventricular hypertrophy in MCT*-*treated minipigs.

**Conclusion:**

A chronic pulmonary hypertension model can be successfully established in young minipigs at six weeks after MCT injection. These minipig models exhibited features of pulmonary arterial hypertension that can be evaluated by both invasive (RHC) and noninvasive (echocardiography) measurements, and may be used as an easy and stable tool for future studies on pulmonary hypertension.

## Introduction

Pulmonary hypertension (PH) in humans, defined by a mean pulmonary artery pressure (PAP) greater than 25 mmHg at rest as measured by right heart catheterization (RHC) [1]–[3] is a syndrome in which pulmonary arterial obstruction increases local vascular resistance, leading to hypertrophy and failure of the right ventricle (RV), or even death [4]. In laboratory studies, monocrotaline (MCT)*–*and hypoxia*–*exposed mice [5] and rats [6] have been so far commonly used animal models of PH. Although these models have undoubtedly contributed to understanding of the pathological process of pulmonary hypertension [7], rodents are too small in size as an animal model for accurate assessment of cardiopulmonary hemodynamics or use in transthoracic echocardiography as a noninvasive approach to measure the systolic pulmonary artery pressure and to diagnose PH as in humans [8], [9]. In other laboratory animals such as rabbits [10]–[12], lambs [13] and dogs [14], PH models have been established by medication (MCT) or cardiopulmonary bypassing. However, PH modeling in these animals either requires skillful manipulations, or does not satisfactorily allow for a full-spectrum noninvasive measurement. On the other hand, pig is a readily available mammal that appears more anatomically similar to humans. Here we demonstrate a study based on our hypothesis that a novel model of pulmonary hypertension can be easily established in young minipigs [15], which enables both invasive and noninvasive measurements, and hence facilitates future studies on mechanisms of pulmonary vascular remodeling and clinical treatment of chronic PH.

## Materials and Methods

### Animal Treatment

The study protocol was approved by the Animal Care Committee at the Southern Medical University (Guangzhou, China). Twenty-four Tibet minipigs (5.0–6.0 kg in weight and 8 weeks of age) were purchased from the Laboratory Animal Center of Southern Medical University and used for this study. All animals were housed individually in clean stainless steel cages in an animal room set at 23*–*29°C and 55*–*70% relative humidity under a natural day/night cycle. They were provided with regular food, free access to water, and humane care in compliance with the Guides for Care and Use of Laboratory Animals in Guangdong Province China.

After four days of acclimatization, the minipigs were randomized into two groups, namely, the MCT group (n = 12) and the control group (n = 12). Each minipig in the MCT group received a single dose of intraperitoneal injection with MCT (12.0 mg/kg, Chengdu Mansite Pharmaceutical Co., LTD., China) in 75% alcohol (final MCT concentration: 25 mg/ml). The control group received injection of placebo (75% alcohol) via the same route and in the equivalent volume as used in the MCT group. The animals were then returned to their cages and given standard pig chow twice daily and tap water ad libitum for the duration of the study. The study period ranged from injection of MCT or placebo (day 1) to day 42.

### Echocardiographic Studies

On day 42, the animals were anesthetized with pentobarbital sodium (10 mg/kg, i.m.) after intramuscular injections of diazepam (6 mg/kg, i.m., Tianjin Jinyao, China) over the dorsum of the neck. Prior to this procedure, the animals had been kept fasting for 8 hours. The anesthesia was maintained throughout all measurements. The chest and four limbs of each animal were shaved for conventional transthoracic echocardiography which included M-mode and Doppler echocardiographic studies by using a commercial ultrasound scanner (Toshiba Medical Systems Corp., Xario, Japan). M*–*mode and Doppler tracings were recorded at a sweep speed of 200 mm/s. M-mode measurements were conducted according to the recommendations as described elsewhere [16]. All measurements were completed by a designated sonographer who was technically experienced and blinded to the group allocation. Two other senior sonographers reviewed the ultrasound images independently in a blind manner. In cases of any disagreement, consensus was reached between the two reviewers, or with the intervention of a third reviewer, if needed. All measurements represented the means of three cardiac cycles [17].

#### M-mode echocardiography

From all minipigs, the M-mode motion curve of the pulmonary valve was obtained, along with other parameters related to the left ventricle (LV) including the left ventricular shortening fraction (FS %), mitral valvular E*–*F slope (MVEF) and the left ventricular ejection fraction (EF %) [18].

#### Doppler echocardiography

The Doppler frequency spectrum of pulmonary outflow was recorded in the parasternal view at the level of the aortic valve. The parameters of the right heart on Doppler frequency spectrum were measured, including heart rate, aortic valve flow peak velocity (AV_max_), pulmonary valve flow peak velocity (PV_max_), peak velocity of pulmonary valve regurgitation (PRV_max_), peak velocity of tricuspid regurgitation (TRV_max_), pulmonary arterial systolic pressure (PASP) defined as tricuspid regurgitation pressure gradient plus right atrial pressure (RAP), and pulmonary arterial diastolic pressure (PADP) defined as pulmonary valve regurgitation pressure gradient. To observe the tricuspid regurgitation (TR), the transducer was aligned to the right atrium, and pulmonary valve regurgitation (PR) was observed from the right ventricular outflow tract. The RAP was estimated to be 5 mmHg, 10 mmHg, and 15 mmHg when the tricuspid regurgitation jet maximum area was visually <20%, 20–50% or >50% of the right atrial maximum area, respectively [19], [20]. Pulmonary artery mean pressure (PAMP) was estimated by using the formula PAMP = [PADP +1/3 (PASP *–* PADP)] [21]. The diastolic right atrium*–*right ventricle pressure gradient (RA*–*RVP), pulmonary artery valve flow acceleration time (PAAT, time from the onset of pulmonary flow to peak velocity by pulsed-wave Doppler recording), pulmonary artery valve ejection time (ET, time interval between the onset and end of the systolic flow velocity), and pulmonary artery valve pre-ejection phase (PEP, time from the onset of QRS wave to the onset of pulmonary valve flow on Doppler echocardiogram) were recorded [22], [23].

### Invasive Measurements by Right Heart Catheterization

**Figure 1:**
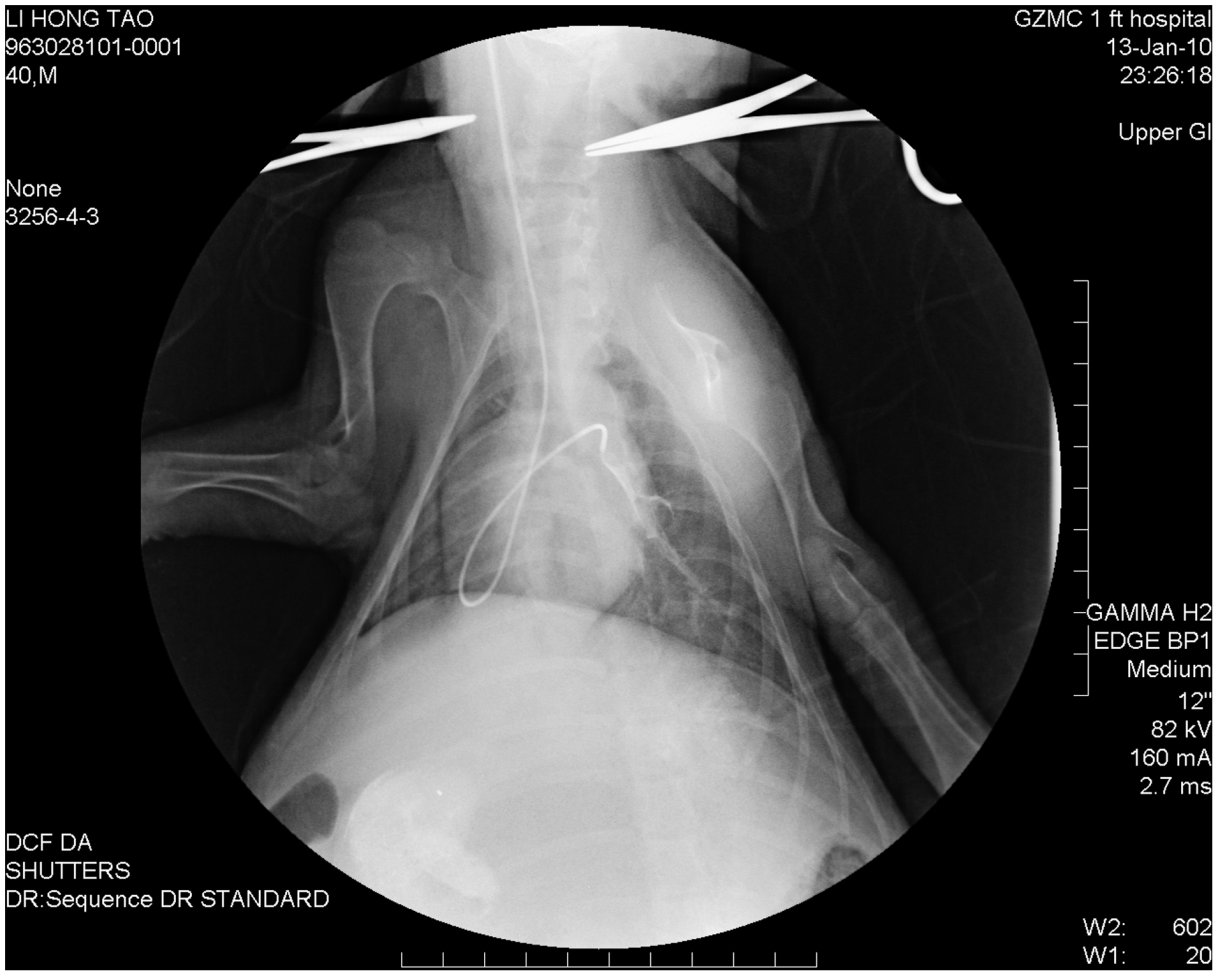
X-ray visualization for positioning the catheter tip in the main pulmonary artery of a minipig.

After echocardiographic studies, the minipigs were immobilized in the supine position and breathing spontaneously. Right heart catheterization (RHC) was performed by a well-trained investigator (Li HT) according to descriptions in the literature. Briefly, an incision was made at the right side of the animal’s neck and dissection was performed to expose the right internal jugular vein. After placement of a guidewire in the vein by using Seldinger technique, a 5*–*French hydrophilic*–*coated vascular sheath (Cordis Corp., Mexico) was placed in this vein [24]. Then a 4*–*French curved*–*tipped angiographic catheter (Cordis Corp., Mexico), pre*–*filled with 1% heparinized saline, was finally inserted from the vascular sheath into the main pulmonary artery [25],[26]. The entire procedure was monitored under fluoroscopic guidance (Toshiba Medical Systems Corp., DBX*–*6000A, Japan) (Figure 1). Then the catheter was connected to a pre-calibrated pressure transducer which in turn was linked to a MedLab biological signal collecting and processing system (V6.0 Nanjing Meiyi Technological Co., China). After instrumentation was completed, the following signals as hemodynamic measurements were continuously recorded on a computer for subsequent analysis in each experiment: systolic pulmonary arterial pressure (sPAP), diastolic pulmonary arterial pressure (dPAP) and mean pulmonary arterial pressure (mPAP).

### Index of Right Ventricular Hypertrophy

After hemodynamic measurements were obtained, animals were euthanized with an overdose of pentobarbital and 10% KCI [27], [28]. Afterwards, the heart and lungs were removed en bloc for morphometric measurements. The right ventricle was dissected from the left ventricle and interventricular septum, and was weighed separately. The right ventricular weight index as a measure of right ventricular hypertrophy, was expressed as the ratio of right ventricular (RV) weight to the combined weights of the left ventricle and interventricular septum (LV+S) [29], [30].

### Pathological Study

The lungs of minipigs were harvested and dissected off adjacent tissues. Tissue blocks were sliced orthogonal to the pulmonary artery, and then prepared by fixation with 4% formaldehyde phosphate buffer for 48 h, followed by alcohol dehydration and paraffin embedding. Microtomed tissue sections (5 µm thickness) were stained with hematoxylin and eosin before routine optic microscopy [30], [31].

### Statistical Analysis

All values are given as the mean ± standard deviation (SD) and analyzed with analysis of variance (ANOVA) and *t* tests by using the Statistical Package for Social Sciences (Ver13.0, SPSS Inc., Chicago, IL, USA). A *p*<0.05 was considered significant. Regression analysis was used to compare the mPAP values to PAMP values and the ET values include two groups by using SAS9.2 (SAS Institute Inc., Cary, NC, USA).

## Results

During the study period, none of the animals died or showed significant signs related to right heart failure, such as dyspnea or peripheral edema. There was no difference in body weight between the two groups (*P = 0.47*).

### Findings of Echocardiographic Studies

**Figure 2:**
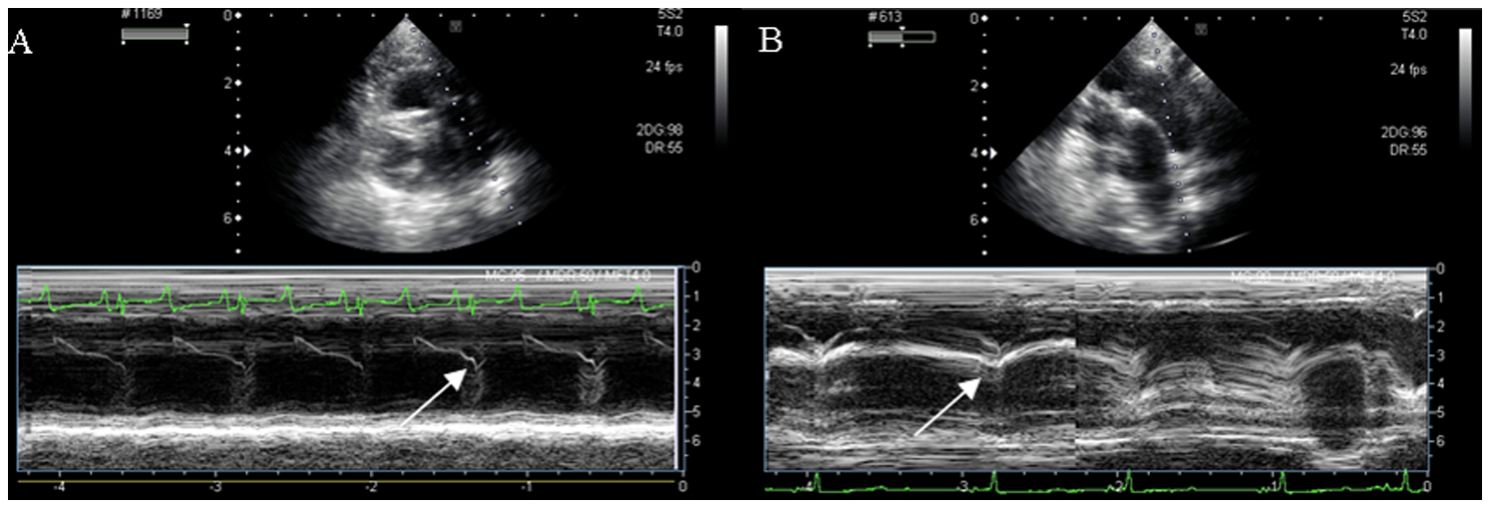
M-mode imaging in all minipigs at 6 weeks after MCT or placebo injection. Panel A: *a-*wave in the control group. Panel B: The V-shaped signals with no *a-*wave in the MCT group (arrows).

M-mode echocardiography indicated comparable systolic and diastolic functions of the left ventricle between the two groups (Table 1). The pulmonary valve curve in the control group appeared as usual signals with *a–*waves, in contrast to the V-shaped signals with diminishing of *a-*waves in the MCT group, a typical feature of pulmonary hypertension (Figure 2).

**Table 1:** Parameters by echocardiography and by right heart catheterization in minipigs on day 42 of the study.

	MCT group	Control group	*P-*value
Body weight(kg)	9.8±1.1	9.2±2.3	0.47
AV_max_ (cm/s)	75.9±3.8	78.0±4.5	0.052
PV_max_ (cm/s)	68.2±5.0	82.9±4.4	<0.0001
TRV_max_ (cm/s)	270.9±25.8	214.5±10.3	0.0002
PRV_max_ (cm/s)	205.6±14.6	167.7±8.2	<0.0001
PASP (mmHg)	41.0±6.3	24.2±2.7	<0.0001
PADP (mmHg)	16.9±2.4	11.2±1.1	<0.0001
PAMP (mmHg)	24.9±3.7	15.6±1.6	<0.0001
RA-RVP (mmHg)	2.0±0.4	2.6±0.2	0.006
PEP (s)	0.06±0.01	0.04±0.00	0.001
PAAT (s)	0.06±0.01	0.08±0.02	0.038
ET (s)	0.43±0.04	0.52±0.07	0.024
Heart rate (beats/min)	97.7±18.5	104.2±20.3	0.269
FS (%)	41.3±3.9	40.1±6.2	0.658
EF (%)	74.7±4.2	73.1±6.6	0.579
MVEF (mm/s)	89.6±16.7	101.8±27.5	0.321
sPAP (mmHg)	33.97±1.66	22.99±1.70	<0.01
dPAP (mmHg)	19.94±2.46	11.32±1.07	<0.01
mPAP (mmHg)	24.62±1.38	15.19±0.70	<0.01
RV weight (g)	11.0±0.5	8.6±0.3	0.0002
(LV+S) weight (g)	30.4±3.1	30.3±2.3	0.9
RV/(LV+S)	0.36±0.16	0.28±0.13	0.044

MCT = monocrotaline; AVmax = aortic valve flow peak velocity; PVmax = pulmonary valve flow peak velocity; PR = pulmonary valve regurgitation; PRVmax = peak velocity of pulmonary valve regurgitation; TR = tricuspid regurgitation; TRVmax = peak velocity of tricuspid regurgitation; PASP = pulmonary arterial systolic pressure (estimated by echocardiography); PADP = pulmonary arterial diastolic pressure (estimated by echocardiography); PAMP = pulmonary artery mean pressure = [PADP +1/3 (PASP-PADP)]; RAP = right atrial pressure (estimated by echocardiography); RA-RVP = The diastolic right atrium-right ventricle; PEP = pulmonary artery valve pre-ejection phase; PAAT = pulmonary artery valve flow acceleration time; ET = pulmonary artery valve ejection time; FS% = shortening fraction of the left ventricle; MVEF = mitral valvular EF slope rate; EF% = left ventricular ejection fraction; RHC = right heart catheterization; sPAP = systolic pulmonary arterial pressure measured by RHC; dPAP = diastolic pulmonary arterial pressure measured by RHC; mPAP = mean pulmonary arterial pressure measured by RHC;RV = the right ventricle;LV+S = the left ventricle and interventricular septum.

**Figure 3:**
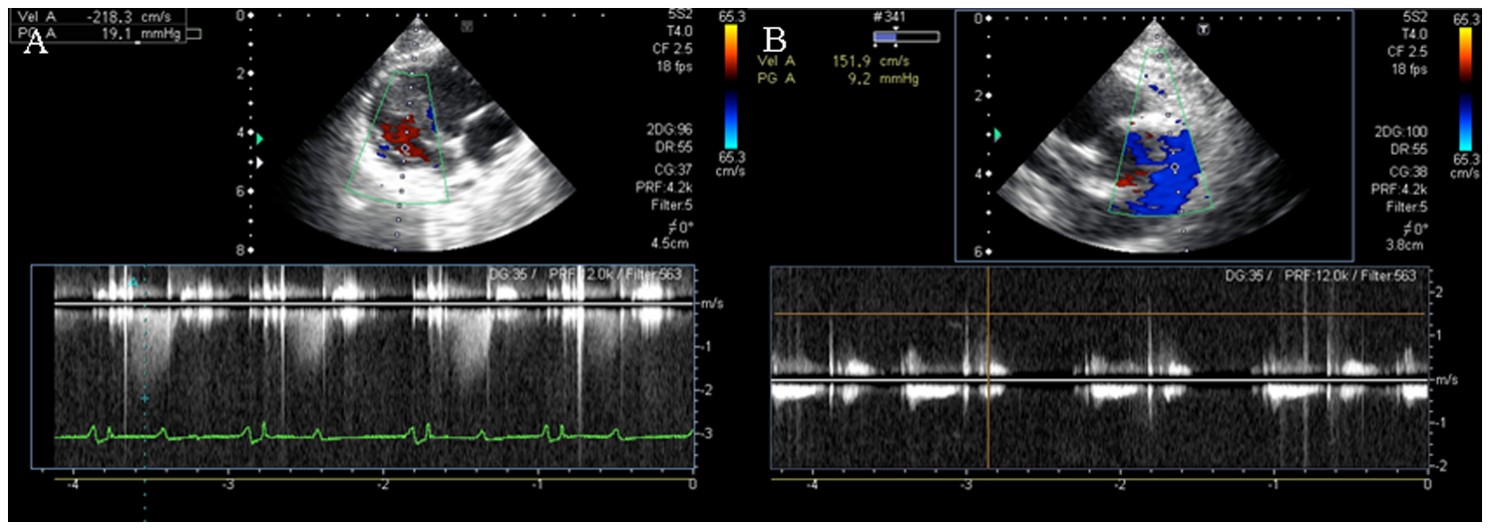
Measurements of the velocity and pressure gradient of the tricuspid regurgitation (Panel A) and pulmonary valve regurgitation (Panel B) using continuous-wave Doppler in a minipig at 6 weeks after placebo injection in the control group.

At pulsed*–*wave Doppler echocardiography, there was no difference in AV_max_ between the two groups (*P*>0.05), but the PV_max_ was significantly lowered in the MCT group than in the control group (*P*<0.0001) (Table 1). Continuous-wave Doppler echocardiography revealed regurgitation at both tricuspid and pulmonary valves in all minipigs (Figure 3). However, the values of TRV_max_ and PRV_max_, as the estimates of pulmonary artery pressures, were higher in the MCT group than those in the control group (*P = *0.0002; *P*<0.0001) (Table 1). The MCT group also showed significantly greater PASP (41.0±6.3 mmHg *vs* 24.2±2.7 mmHg), PADP (16.9±2.4 mmHg *vs* 11.2±1.1 mmHg) and PAMP (24.9±3.7 mmHg *vs* 15.6±1.6 mmHg) as compared with the control group (all *P*<0.001) (Table 1). Notably, the PASP met the diagnostic criteria for primary pulmonary hypertension in humans. In addition, the diastolic right atrium-right ventricle pressure gradients (RA*–*RVP) in the MCT group were lower than that in the control group (2.0±0.4 mmHg *vs* 2.6±0.2 mmHg, *P = *0.006).

**Figure 4:**
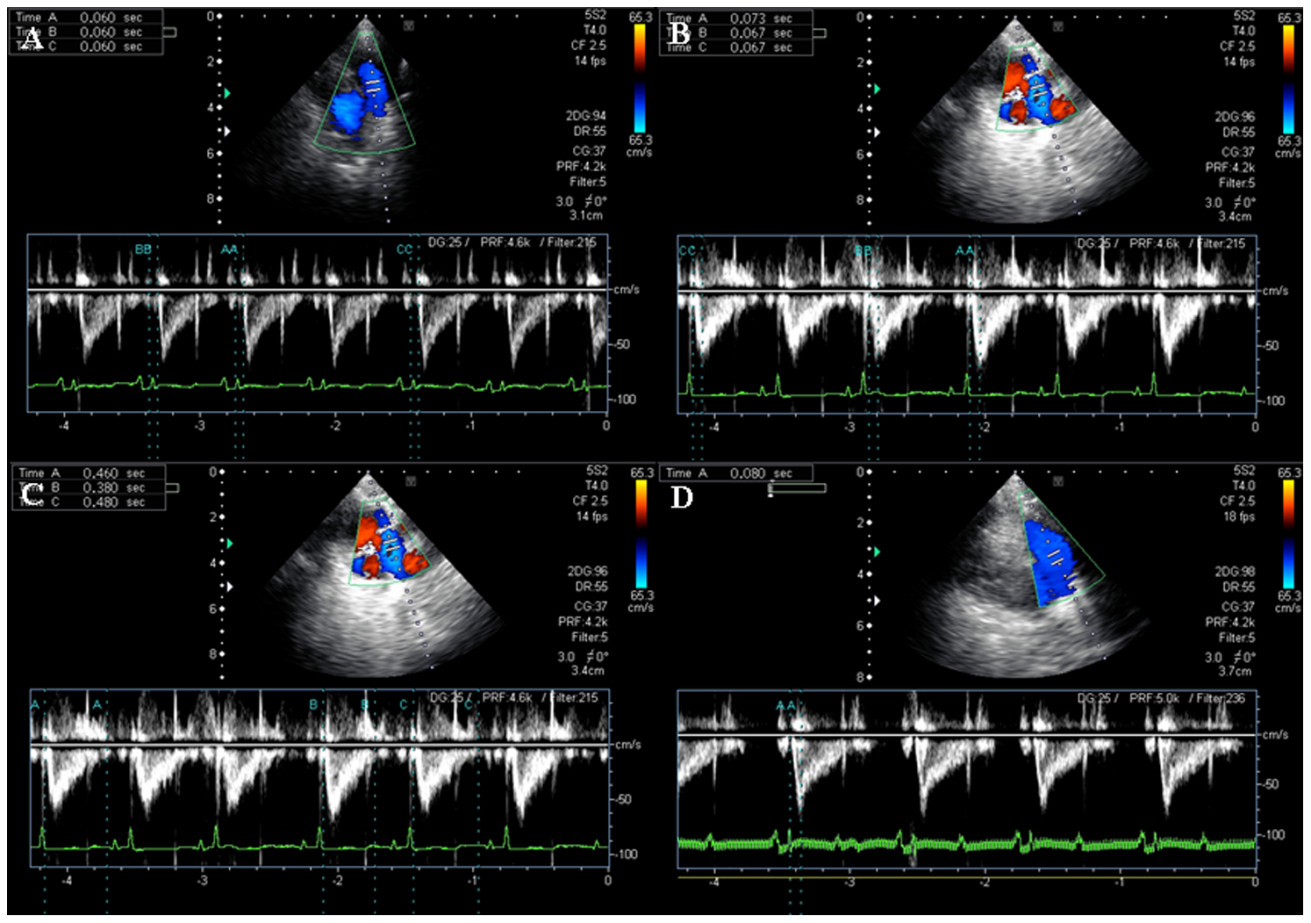
Measurements of pulmonary flow using pulsed-wave Doppler in a minipig at week 6 of the study. Panel A: pulmonary artery valve pre-ejection phase (PEP, time from the onset of QRS wave to the onset of pulmonary valve flow on Doppler echocardiogram) in MCT group; Panel B: pulmonary artery valve flow acceleration time (PAAT, time from the onset of pulmonary valve flow to peak velocity by pulsed-wave Doppler recording) in MCT group; Panel C: pulmonary artery valve ejection time (ET, time interval between the onset and end of the systolic flow velocity) in MCT group and Panel D: PAAT in the control group, measured based on the QRS wave.

Of either group, the blood flow of pulmonary arteries in the minipigs displayed triangular, dagger–shaped Doppler signals (Figure 4). Compared with the control group, the MCT–treated minipigs had longer PEP (0.06±0.01 s vs 0.04±0.00 s, P = 0.0008), shorter PAAT (0.06±0.01 s vs 0.08±0.02 s, P = 0.038) and ET (0.43±0.04 s vs 0.52±0.07, P = 0.024), indicating weakened right heart function (Table 1). There were no significant differences between the two groups in parameters of left ventricular function, such as heart rate (P = 0.269), FS% (P = 0.658), EF% (P = 0.579) and MVEF (P = 0.321) (Table 1).

### Findings of Hemodynamic Study

**Figure 5:**
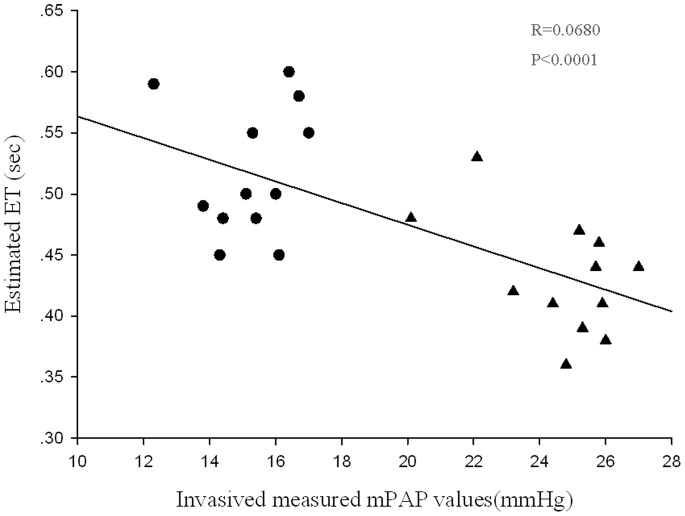
Linear regression plot showing a negative correlation between the mPAP values measured by invasive right heart catheter and the ET values estimated by echocardiography (R = 0.680, P<0.0001). mPAP = mean pulmonary arterial pressure; ET = pulmonary artery valve ejection time. •,the control group;▴,MCT group.

Results of pulmonary artery pressure in the two groups of minipigs at six weeks after MCT or placebo injection are also shown in Table 1. Compared with the control group, the minipigs treated with MCT experienced higher sPAP (34.0±1.7 mmHg vs. 23.0±1.7 mmHg), dPAP (19.9±2.5 mmHg vs. 11.3±1.1 mmHg) and mPAP (24.6±1.4 vs. 15.1±0.8 mmHg) at six weeks after the injection (all *P*<0.01), which fulfilled the clinical criteria for identification of primary pulmonary hypertension in humans. Linear regression shows the positive linear correlation (R = 0.974, P<0.0001) between the mPAP values and the PAMP values. Figure 5 shows a negative correlation (R = 0.680, P<0.0001) between the mPAP values and the ET values.

### Index of Right Ventricular Hypertrophy

There were no significant differences between the two groups in the body weight and (LV+S) weights, The ratio of RV weight to (LV+S) weights in the MCT group was significantly higher compared with the control group (0.36±0.16 *vs.* 0.28±0.13, *P = 0.044*<0.05) (Table 1), suggesting the development of RV hypertrophy as a consequence of increased pulmonary pressure in minipigs injected with MCT.

### Findings of Pathological Changes

**Figure 6:**
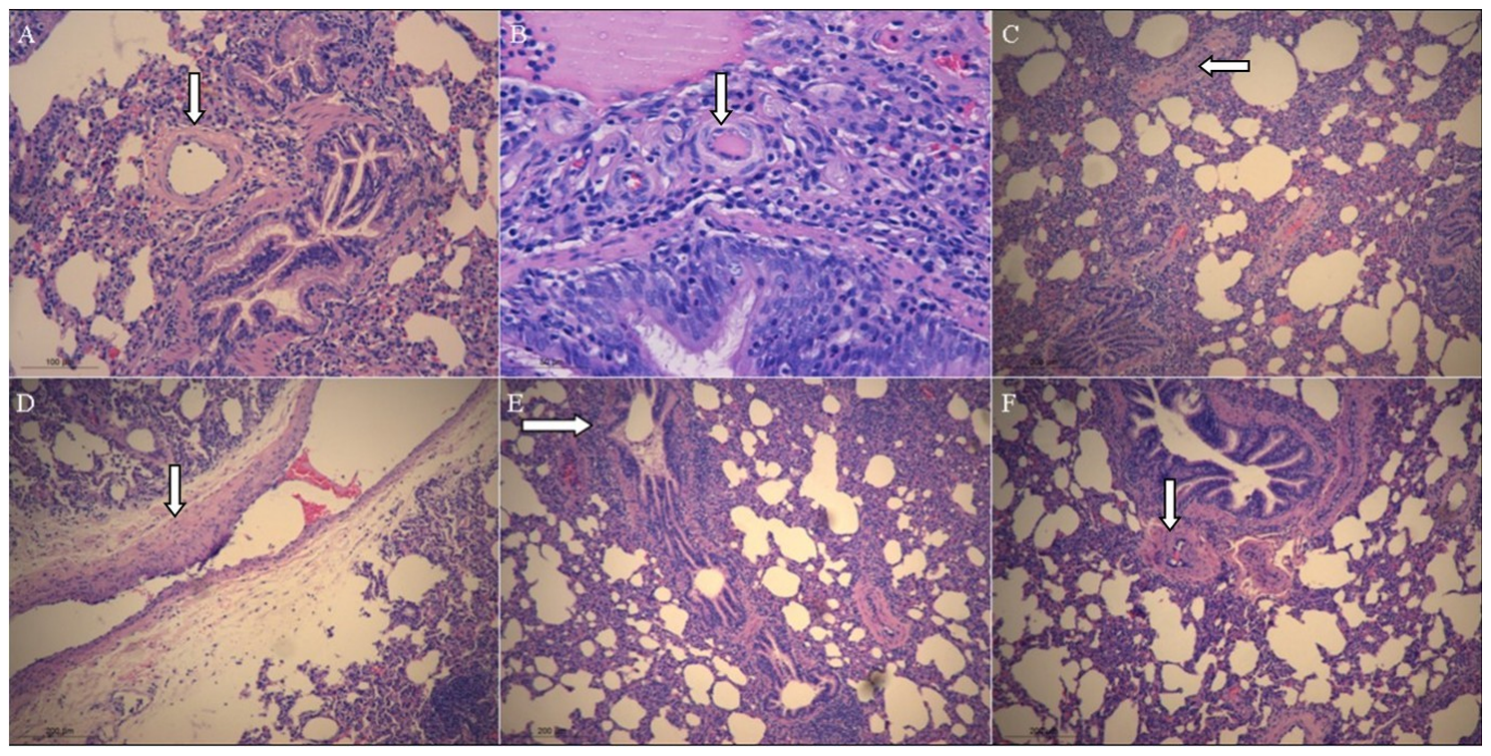
Typical microscopic views of pulmonary hypertension (PH) in a 14-week-old pig at 6 weeks after injection of monocrotaline; Panel A, control group; Panels B-F, MCT group. Panel A: Typical microscopic view of a small muscular pulmonary artery (arrows) in a 14-week-old minipig from the control group. The lumen of normal pulmonary artery is surrounded by a uniform thin wall with no fibrosis or inflammatory cell infiltration (Hematoxylin and Eosin staining; original magnification:×200; scale bar = 100 µm); Panel B: The vessel lumen is narrowed, occluded, twisted or deformed with formation of plexiform lesions (arrows) as a result of proliferating and hypertropic endothelial and smooth muscle cells, intimal fibrosis and accompanying hyalinization (Hematoxylin and Eosin staining; original magnification:×400; scale bar = 50 µm). Panel C: Pulmonary vascular remodeling in the lung tissue. There was intimal thickening accompanied with plexiform lesions (arrows) (Hematoxylin and Eosin staining; original magnification:×100; scale bar = 200 µm). Panel D: Uneven thickening of the pulmonary arterial wall (arrows). (Hematoxylin and Eosin staining; original magnification:×100; scale bar = 200 µm); Panel E: The presence of large amount of mucus (arrows) in bronchioles led to luminal obstruction and lymphocyte infiltration. In the periphery of lungs, congestion of capillaries, thickening of interstitium and focal lymphocyte infiltration were observed (Hematoxylin and Eosin staining; original magnification:×100; scale bar = 200 µm). Panel F: Parabronchus arteries (arrows) were largely occluded owing to extensive neointimal proliferation and medial hypertrophy accompanied with hyalinization (Hematoxylin and Eosin staining; original magnification:×100; scale bar = 200 µm).

The lumen of the pulmonary artery in the control group was surrounded by a uniform thin wall with no fibrosis or inflammatory cell infiltration (Figure 6 A). In contrast, the lung tissues from the MCT group exhibited a clear picture of pulmonary vascular remodeling accompanied with proliferation and hypertrophy of endothelial and smooth muscle cells. Varying degrees of intimal fibrosis and hyalinization were also noted. In addition, the vessel lumen was narrowed, occluded, twisted or deformed with formation of plexiform lesions (Figures 6 B and C). On a large number of sections, the walls of pulmonary artery were apparently uneven in thickness (Figure 6 D). The presence of large amount of mucus in bronchioles led to luminal obstruction and lymphocyte infiltration. In the periphery of lungs, congestion of capillaries, thickening of interstitium and focal lymphocyte infiltration were observed (Figure 6 E). Juxtabronchial arteries and veins were largely occluded owing to extensive neointimal proliferation and medial hypertrophy accompanied with hyalinization (Figure 6 F). These histological changes were similar to those observed in patients with PH [32].

## Discussion

Animal models of pulmonary hypertension are important for all-round understanding of this condition in humans. While laboratory animals such as rats, mice, rabbits, dogs and lambs have been used to model PH, porcine models can be valuable in studies focusing on pulmonary hemodynamics since they appear more anatomically similar to humans. Previous efforts to establish the PH model in pigs frequently involved vascular anastomosis [33] or exposure to hypoxia [34]. Such models seemed more eligible to mimic an acute rather than a chronic process of PH [35]–[40]. Therefore, experiments on attempts to develop reliable and stable pig models of chronic PH in shorter period and easier approach would be of significant scientific interest, but have rarely been published so far, to the best of our knowledge.

Our previous study showed that minipigs (4*–*5 month*–*old) developed PH at 8 weeks after injection of MCT at a lower single dose (10.0 mg/kg) [41]. In that study, we measured invasively only one parameter (PAP) by right heart catheterization. The mPAP was 15.2±0.7 mmHg at baseline and 21.8±1.7 mmHg at 6 weeks. Obviously, the modeling of PH was not successful until more weeks later (week 8, 25.6±4.9 mmHg). Moreover, some difficulties arose as to capturing and immobilizing the animals before the experiment, since the minipigs had gained in body weight up to 20 kg at 8 weeks. Given that younger minipigs with rapidly maturing lungs may be more susceptible to MCT [42], we selected 2*–*month*–*old pigs for establishing the PH model in the present study. The results showed that intraperitoneal use of MCT (12.0 mg/kg) was associated with significantly higher PAP at 6 weeks after the injection compared with controls, as revealed by invasive measurement with right heart catheterization (RHC). In consistent with clinical PH in humans, the sPAP was 34.0±1.7 mmHg in the MCT group. Moreover, the index of right ventricular hypertrophy, a major indicator of PH, was elevated in the MCT group compared with the control group. Such an elevation was in line with the high level of PAP which adds to the burden of right ventricular ejection and thereby induces the right ventricle hypertrophy.

Pathological study also confirmed development of PH in MCT*–*treated minipigs of the present study. Compared with placebo, injection of MCT (12.0 mg/kg) in young pigs was associated with extensive vascular remodeling in pulmonary arteries at 6 weeks later, including medial hypertrophy, neointimal proliferation, and interstitial thickening (Figure 2). These histological changes were similar to as found in patients with PH [3], suggesting that the minipig models could reproduce the human disease better than rats in which the vascular remodeling was limited merely to medial hypertrophy [7].

Although RHC is recognized as the “gold standard” for identification of PH, the time-consuming healing of blood vessel wounds may not allow for repetitive or continuous observation of pulmonary hypertension in pigs with RHC. For this goal, transthoracic echocardiography is one of common modalities for observing the progression of PH in humans [34]. Doppler echocardiography of the tricuspid and pulmonary valves has been recommended by the European Society of Cardiology as one of the first steps in evaluation of the patient with suspected PH [1]. Although not the “gold standards” for identification of prognostic determinants in patients with PH, qualitative parameters, such as changes in pulmonary flow characteristics, and TR and PR, are established echocardiographic features of pulmonary hypertension in humans. Echocardiographically derived PAP has been reported to well correlate (r = 0.73–0.94) with invasive measurements in humans [35], [36]. The velocity of the tricuspid regurgitation jet measured with continuous-wave Doppler echocardiography as a surrogate of pulmonary artery systolic pressure is the mainstay of assessing the severity of PH. These measurements, however, require the presence of right*–*sided valvular regurgitation in sufficiently large volume to produce Doppler signals that allow for accurate measurement of the peak TR velocity [43]. Based on the TR, systolic right ventricular*–*to*–*right atrial pressure gradients may be calculated by using the modified Bernoulli equation (pressure gradient = 4×[velocity in meters per second]^2^ to the peak tricuspid or pulmonic regurgitant jet velocity, respectively) [44]. To obtain an estimated PASP, these pressures are added to the estimated RAP of pigs. The estimation of RAP value is based on the ratio of maximum area of TR to the maximum area of right atrium[>50%, 15 mmHg; 50%*–*20%, 10 mmHg; <20%, 5 mmHg] [19]. At the same time, diastolic pulmonary artery-to-right ventricular pressure gradients are calculated based on the PR, which is equal to the PADP (diastolic blood pressure of the right atrium is equal to zero when there is no congestive heart failure).

Because minipigs and human beings are similar in anatomic structure and body type [45], parameters of pulmonary arterial pressure in the animal models can be readily assessed by means of echocardiography. Our assessment revealed that the PASP, PADP and PAMP values, like the sPAP, dPAP and mPAP in invasive measurements (RHC), also met the clinical criteria of primary pulmonary hypertension. Linear regression shows that there was a significant and positive correlation between the PAMP values and the mPAP values (R = 0.974, P<0.0001).

The PASP, PADP and PAMP values in the minipigs of MCT group were obviously greater than those in the controls (*P*<0.0001). The differences between PASP and sPAP as well as between PADP and dPAP might arise from the errors in subjective estimation of RAP in the pigs. The diastolic right atrium-right ventricle (RA*–*RVP) pressure gradients in the MCT group were lower than that in the control group (*P = *0.006), which was consistent with the higher diastolic right ventricle pressure in the MCT group as a result of right ventricular hypertrophy.

The time interval in the pulmonary blood flow frequency spectrum is also an important index for pulmonary hypertension on Doppler Echocardiography. In the present study, the MCT*–*treated minipigs had longer PEP but shorter PAAT and ET, as compared with the controls. This may be explained by the stronger contraction of RV, and hence more time needed than usual, to open the pulmonary valve under elevated pulmonary pressure. Because of the delayed opening of the pulmonary valve, PEP would be prolonged. In the meanwhile, the premature closure of pulmonary valve would give rise to the shortened ET. Regression analysis also shows that there was a negative correlation between the ET and the mPAP (R = 0.680, P<0.0001).

In two experiments, the systolic and diastolic functions of the left ventricle were reduced in MCT*–*treated rats [7], [46]. Our study did not show significant difference between the two groups regarding the heart rates of the pigs, nor in other indexes about the contraction and diastolic function, such as FS%, EF% and MVEF. As in other clinical observations of PH [7], the left ventricular function of pigs was not reduced in MCT*–*treated pigs.

The M*–*mode signals of pulmonary valve had *a–*waves in the control group but not in the MCT group, *a–*wave disappeared. This may stem from the pulmonary hypertension that caused the pulmonary valve to close prematurely at the systolic phase, and therefore has a specific value for clinical diagnosis.

Taken together, these findings confirmed the successful establishment of a chronic PH model in minipigs at six weeks after a single-dose MCT injection. These minipig models exhibited features of pulmonary arterial hypertension that can be evaluated by not only invasive (RHC) but also noninvasive (echocardiography) measurements. Importantly, the echocardiographic parameters in these minipig models were very close to the clinical criteria for identification of PH. To a significant extent, echocardiography can be used as an alternative to right heart catheterization to predict PASP in minipig PH models.

Several limitations in this study should be acknowledged. Firstly, the invasive measurement of pulmonary pressure was performed using an angiocatheter. Future studies would ideally use a Swan-Ganz catheter by which the wedge pressure and cardiac output can be measured simultaneously. Secondly, we did not perform sequential echocardiographic measurements in the present study, because our study protocol was designed to performed invasive and non-invasive measurements simultaneously. Further studies on minipigs with sequential echocardiography would be helpful to clarify the shortest time with the single dose of MCT as in this study to develop PH, or to find out lowest dose of MCT to develop PH in a given duration.

Nevertheless, the minipig model investigated in the present study can be an easy and stable tool for continuous study on the mechanisms of pulmonary vascular remodeling and the clinical treatment of chronic PH.
